# MIT-001 Restores Human Placenta-Derived Mesenchymal Stem Cells by Enhancing Mitochondrial Quiescence and Cytoskeletal Organization

**DOI:** 10.3390/ijms22105062

**Published:** 2021-05-11

**Authors:** Won Dong Yu, Yu Jin Kim, Min Jeong Cho, Gi Jin Kim, Soon Ha Kim, Myung Joo Kim, Jung Jae Ko, Jae Ho Lee

**Affiliations:** 1 Department of Biomedical Science, College of Life Science, CHA University, Pocheon 11160, Korea; manseo60@gmail.com (W.D.Y.); mjjj0725@gmail.com (M.J.C.); gjkim@cha.ac.kr (G.J.K.); 2 CHA Fertility Center, Seoul Station, Hangang-daero, Jung-gu, Seoul 04637, Korea; yj_kim@chamc.co.kr; 3 Mitoimmune Therapeutics Inc., Gangnam-gu, Seoul 06253, Korea; shakim@mitoimmune.com

**Keywords:** mesenchymal stem cells, inflammation, homeostasis, mitochondria, cytoskeleton, MIT-001

## Abstract

Inflammation is a major cause of several chronic diseases and is reported to be recovered by the immuno-modulation of mesenchymal stem cells (MSCs). While most studies have focussed on the anti-inflammatory roles of MSCs in stem cell therapy, the impaired features of MSCs, such as the loss of homeostasis by systemic aging or pathologic conditions, remain incompletely understood. In this study, we investigated whether the altered phenotypes of human placenta-derived MSCs (hPD-MSCs) exposed to inflammatory cytokines, including TNF-α and IFN-γ, could be protected by MIT-001, a small anti-inflammatory and anti-necrotic molecule. MIT-001 promoted the spindle-like shape and cytoskeletal organization extending across the long cell axis, whereas hPD-MSCs exposed to TNF-α/IFN-γ exhibited increased morphological heterogeneity with an abnormal cell shape and cytoskeletal disorganization. Importantly, MIT-001 improved mitochondrial distribution across the cytoplasm. MIT-001 significantly reduced basal respiration, ATP production, and cellular ROS levels and augmented the spare respiratory capacity compared to TNF-α/IFN-γ-exposed hPD-MSCs, indicating enhanced mitochondrial quiescence and homeostasis. In conclusion, while TNF-α/IFN-γ-exposed MSCs lost homeostasis and mitochondrial quiescence by becoming over-activated in response to inflammatory cytokines, MIT-001 was able to rescue mitochondrial features and cellular phenotypes. Therefore, MIT-001 has therapeutic potential for clinical applications to treat mitochondrion-related inflammatory diseases.

## 1. Introduction

Inflammation is produced not only by infectious agents such as bacteria and viruses but also by non-infectious causes like intrinsic dysregulation of the immune system [1]. A coordinated series of mechanisms for inflammation contribute to tissue injury, oxidative stress, remodeling of the extracellular matrix, angiogenesis, and fibrosis in specific tissues, suggesting that inflammation is a major cause of acute and chronic defects in several diseases, including arthritis, asthma, atherosclerosis, cancer, dementia, and autoimmune disease [2]. While common immune processes protect against various pathogens, excessive and consecutive levels of inflammation lead to different disease states acting on diverse cell types [3]. In particular, irregular inflammatory responses act on mesenchymal cells, resulting in the development of degenerative disorders [4,5]. However, despite the significance of MSCs in inflammatory responses, therapeutic methods for inflammation-related diseases still have not been completely established in the clinical field. Therefore, in this study, we address the close correlation between MSCs and inflammatory responses to diseases.

Mesenchymal stem cells (MSCs) are multi-potent cells that play a role in tissue homeostasis and regeneration. MSCs have been widely studied for their clinical possibilities in stem cell therapy due to their anti-inflammatory and tissue regenerative capacity [6,7]. However, the age-related effects caused by other factors on MSCs are relatively less understood. Recently, growing evidence has shown that reversal of the age-related phenotype of MSCs could enhance systemic health and longevity [8,9,10,11]. MSCs exhibit a unique metabolic feature called the quiescent state, which refers to a low level of metabolism that relies mainly on glycolysis, rather than mitochondria [12,13]. In practice, MSCs feature immature and inactive mitochondria in contrast to other differentiated cell types, allowing them to maintain their quiescence and homeostasis [13]. Therefore, maintaining the mitochondrial function of MSCs is becoming increasingly important to ensure not only their availability for stem cell therapy but also their regenerative and homeostatic capacity for tissue homeostasis. However, MSCs begin to lose their properties due to numerous factors, including DNA damage, altered intercellular communication, mitochondrial dysfunction, and the accumulation of toxic metabolites both in vivo and in vitro [14,15,16].

There are many known aging mechanisms that cause the above consequences, but one of the most prominent systemic factors is age-related chronic damage, including pro-inflammatory cytokines from an altered immune system. For instance, Romieu-Mourez et al. reported that MSCs suffer senescence through exposure to pro-inflammatory cytokines, such as tumor necrosis factor-α (TNF-α) and interferon-γ (IFN-γ), during aging [17]. Wang et al. further confirmed that TNF-α and IFN-γ act as major inflammatory executers of MSC impairment by synergistically inducing osteogenic differentiation deficiency and malignant transformation via inflammatory signal pathways, such as NFκB/SMAD7, NFκB/c-Fos, and c-Myc [5]. Likewise, numerous studies have shown that exposure to TNF-α and IFN-γ yields the homeostasis and quiescence of MSCs [5,18,19,20]. Nonetheless, the mitochondrial phenotype of TNF-α/IFN-γ-exposed MSCs has yet to be identified, despite the unique and critical mitochondrial properties of MSCs. In addition, although many researchers have reported changes in the cytoskeleton during stem cell maintenance and differentiation [21,22,23], the impaired cytoskeletal organization of MSCs by means of systemic inflammation remains poorly understood.

The novel necrosis inhibitor “MIT-001” (C24H29N3O3S, molecular mass 516.67 Da) is a mitochondrion-targeted ROS scavenger that was initially found to inhibit necrotic cell death. However, several studies have reported that MIT-001 exerts various therapeutic effects on inflammatory or degenerative disease [24,25,26,27]. For instance, Kim et al. reported that MIT-001 (known as NecroX-7) inhibits osteoclast differentiation in LPS-treated bone marrow cells in C57/BL6 mice, thereby significantly reducing degenerative bone loss [25]. Im et al. also demonstrated that NecroX-7 attenuates acute graft versus host disease (GvHD) via reciprocal regulation of type 1 helper T cells and regulatory T cells, with inhibition of TNF, interleukin-6 (IL-6), and TLR4 [27]. Moreover, NecroX compounds have been suggested as promising candidates due to their roles in mitochondrial antioxidant defense to alleviate mitochondrial dysfunction against oxidative stress [26]. Since MSCs are indispensable for tissue homeostasis, including in wound healing, intercellular communication, and immunomodulation, restoring the MSC phenotypes could represent a novel strategy to improve the homeostatic condition of some diseases, such as autoimmune disease. In this study, we demonstrate that MIT-001 can prevent the pro-inflammatory cytokine-exposed hPD-MSCs through the mitochondrial homeostasis, resulting in ameliorated cellular phenotypes and functions.

## 2. Results

### 2.1. MIT-001 Ameliorates the Morphology of TNF-α/IFN-γ-Exposed hPD-MSCs

**Figure 1:**
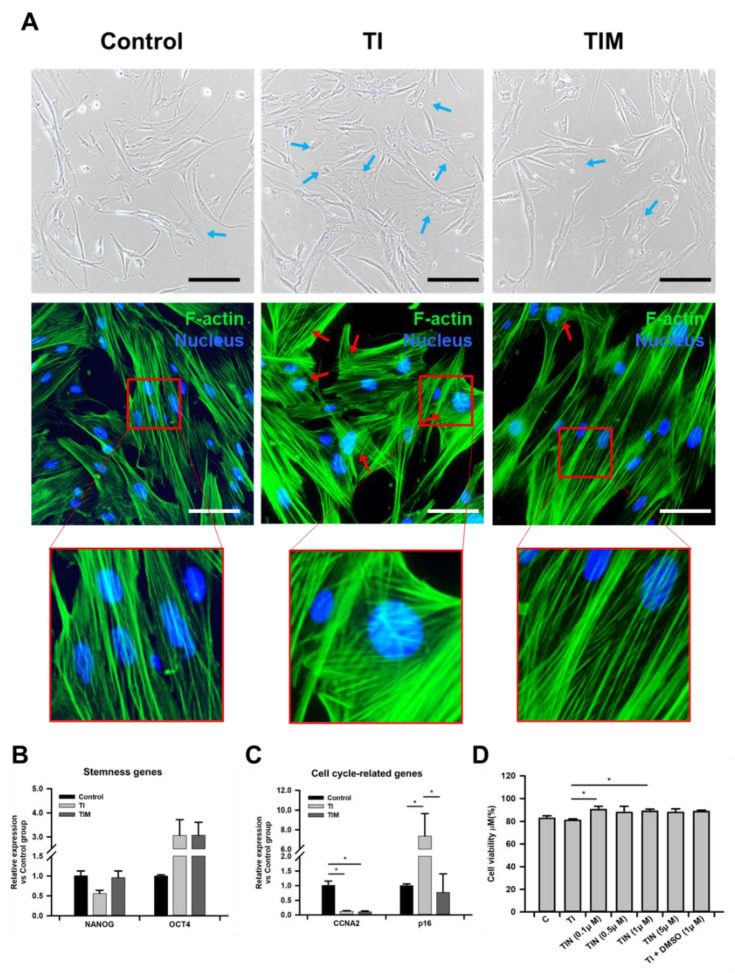
Characterization of hPD-MSCs. (**A**) Representative bright-field images (up) and fluorescence microscopy images of hPD-MSCs (down). Phalloidin for F-actin and Hoechst33342 for the nuclei were used to visualize the morphology of the hPD-MSCs. Blue arrows indicate abnormal MSCs that lost their thin and spindle-like shapes. Red arrows indicate disrupted, crisscrossed, and unorganized microfilaments vertical to the cell axis. Black scale bar, 200 μm, White scale bar, 100 μm. (**B**,**C**) Expression of cell cycle progression (CCNA2), cell cycle inhibitor (P16), and stemness (NANOG and OCT4) genes were determined using quantitative real-time PCR. (**D**) Cell viability was calculated by dividing the cells unstained with Trypan blue by the total number of cells. C: control, TI: TNF-α + IFN-γ, TIM: TNF-α + IFN-γ + MIT-001. Data are presented as the mean ± SEM (*n* = 3 for real-time qPCR data). Statistical significance was determined by a one-way ANOVA. * *p* < 0.05.

We set up TNF-α/IFN-γ-exposed hPD-MSCs with or without MIT-001 and non-treated hPD-MSCs for the control group (passage 11 or 12). In total, 2.5 ng/mL of TNF-α and 25 ng/mL of IFN-γ were exposed to hPD-MSCs for 24 h. Because the morphology of MSC is reportedly a valuable visual marker for MSC health [28], the optimal MIT-001 concentration was determined by observing the morphological recovery effects by concentration in the control, TNF-α/IFN-γ-exposed (TI), and TNF-α/IFN-γ with MIT-001-treated (TIM) hPD-MSCs. As shown in Figure 1A and Figure S1, the control hPD-MSCs displayed slender and normal spindle-like shapes, whereas the TNF-α/IFN-γ-treated hPD-MSCs showed increased morphological heterogeneity, with a flattened, enlarged, and abnormal morphology. In addition, the TNF-α/IFN-γ-exposed hPD-MSCs also exhibited stressed fibers, which refers to unorganized microtubule structures confirmed by F-actin staining (Figure 1A). While non-treated hPD-MSCs presented parallel and organized F-actin structures extending across the long cell axis, TNF-α/IFN-γ-exposed hPD-MSCs demonstrated disrupted and chaotic cytoskeletal organization vertical to the cell axis. Meanwhile, both the abnormal morphology and unorganized microfilaments were markedly ameliorated like the control hPD-MSCs after MIT-001 treatment.

Among the concentrations from 100 to 10 μM, treatment with 1 μM of MIT-001 most efficiently enhanced the morphology of hPD-MSCs exposed to TNF-α/IFN-γ (Figure S1). Therefore, all the subsequent experiments were analyzed with 1 μM of MIT-001. Next, we conducted real-time qPCR to evaluate the expression of the cell cycle and stemness. While TNF-α/IFN-γ treatment significantly decreased cell cycle progressor cyclin A2 (CCNA2) and increased cell cycle inhibitor P16, MIT-001 markedly reduced the cell cycle inhibitor P16 (Figure 1B). Meanwhile, MIT-001 significantly augmented NANOG expression to a similar level to that of the control hPD-MSCs, whereas the expression level of OCT4 was hardly affected (Figure 1C). We also assessed cell viability after TNF-α/ IFN-γ and MIT-001 treatment, as TNF-α and IFN-γ have been reported to induce necroptotic cell death in other cell types [29,30,31], but none of the groups showed an acute cell death rate, as shown in Figure 1D. Collectively, these data demonstrate that MIT-001 improved the cytoskeletal organization and cellular morphology of the TNF-α/IFN-γ-exposed hPD-MSCs.

### 2.2. MIT-001 Remedies the Balance of the Mitochondrial Distribution and Dynamics of TNF-α/IFN-γ-Exposed hPD-MSCs

**Figure 2:**
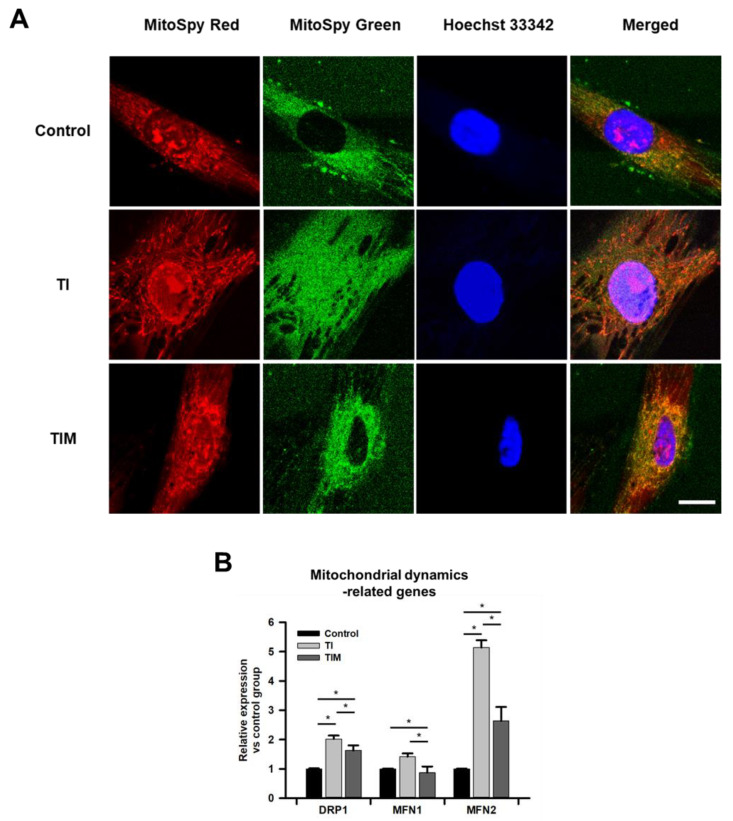
MIT-001 ameliorated mitochondrial function in hPD-MSCs. (**A**) Representative images of control, TNF-α/IFN-γ-exposed, and TNF-α/IFN-γ with MIT-001-treated hPD-MSCs. The mitochondrial membrane potential (red), mitochondrial mass (green), and nuclei (blue) of hPD-MSCs were stained and imaged using confocal microscopy. Scale bar, 20 μm. (**B**) Expression of the mitochondrial dynamics-related genes DPR1, MFN1, and MFN2 was analyzed using real-time qPCR. C: control, TI: TNF-α + IFN-γ, TIM: TNF-α + IFN-γ + MIT-001. Data are presented as the mean ± SEM (*n* = 3 for real-time qPCR data). Statistical significance was determined by a one-way ANOVA. * *p* < 0.05.

MSCs begin to lose their quiescence after exposure to TNF-α and IFN-γ due to a metabolic switch from a quiescent to an activated state. In Figure 2A and Supplementary Video S1, we illustrate how non-treated hPD-MSCs exhibited a uniformly distributed mitochondrial tubular network around the perinuclear site, while TNF-α/IFN-γ-exposed hPD-MSCs showed inconsistently distributed mitochondria reaching the outside of the cytoplasm. However, enhanced consistency of this mitochondrial distribution was observed in MIT-001-treated hPD-MSCs compared to TNF-α/IFN-γ-exposed groups, as shown in Figure 1A. To further investigate these changes in mitochondrial morphology, representative gene expressions for mitochondrial dynamics, including dynamin-related protein 1 (DRP1) for mitochondrial fission, and mitofusion 1 (MFN1), and mitofusion 2 (MFN2) for mitochondrial inner membrane fusion, were analyzed by real-time qPCR. Among the mitochondrial dynamics-related genes, MFN2 was the most strikingly influenced by TNF-α/IFN-γ and MIT-001. TNF-α/IFN-γ-exposed hPD-MSCs revealed a five-fold increase in MFN2 compared to the control hPD-MSCs. However, MIT-001 decreased MFN2 expression by almost 50 percent. Similarly, the expression of DRP1 and MFN1, respectively, demonstrated two-fold and 1.5-fold increases in TNF-α/IFN-γ-exposed hPD-MSCs compared to the non-treated hPD-MSCs, while MIT-001 treatment significantly down-regulated both DRP1 and MFN1 expression by about 20 and 35 percent, respectively. Collectively, these data reveal that MIT-001 could promote the overall landscape of the mitochondrial network in the TNF-α/IFN-γ-exposed MSCs by regulating mitochondrial dynamics and distribution.

### 2.3. MIT-001 Reduced Mitochondrial Oxidative Stress

**Figure 3:**
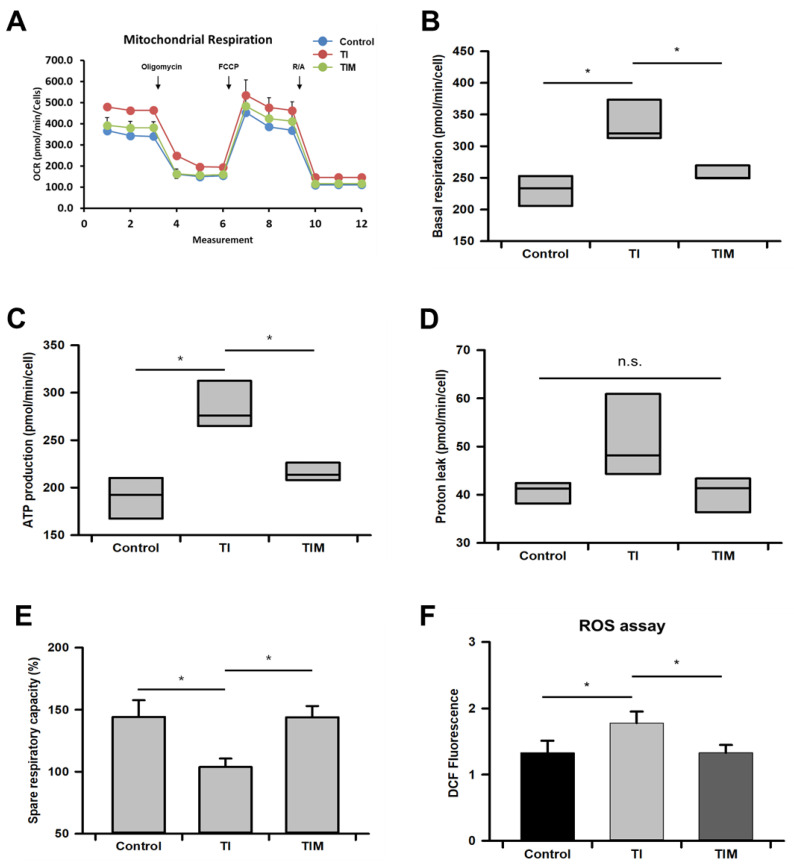
MIT-001 decreased mitochondrial metabolism of hPD-MSCs. (**A**) Comprehensive oxygen consumption rates in hPD-MSCs were measured using an extracellular flux analyzer. R/A: Rotenone/Antimycin. (**B**–**E**) Graphs of the basal respiration (**B**), ATP production (**C**), proton leak (**D**), and spare respiratory capacity (**E**). Cellular ROS levels were determined by measuring the fluorescence intensity of H2DCFDA after 488/555 excitation C: control, TI: TNF-α + IFN-γ, TIM: TNF-α + IFN-γ + MIT-001. Data are presented as the mean ± SEM (*n* = 3). Statistical significance was determined by a one-way ANOVA. * *p* < 0.05.

We measured the oxygen consumption rate of each group of hPD-MSCs to evaluate the mitochondrial metabolic state using a Seahorse XFp extracellular flux analyzer (Figure 3A). While the control hPD-MSCs consumed about 240 pmol/min/cell, exposure to TNF-α and IFN-γ significantly augmented the basal respiration rate by about 100 pmol/ min/cell compared to the control hPD-MSCs (Figure 3B). However, MIT-001 treatment decreased the basal respiration by about 80 pmol/min/cell in the TNF-α/IFN-γ-exposed hPD-MSCs. Similarly, the oxygen consumption for ATP production was about 100 pmol/ min/cell higher in the TNF-α/IFN-γ-exposed hPD-MSCs but was down-regulated by 70 pmol/ min/cell with MIT-001 treatment (Figure 3C). A similar tendency was demonstrated in proton leak with basal respiration and ATP production, but there was no statistical significance between the groups (Figure 3D). In contrast to other values, the spare respiratory capacity (SRC) was 40% diminished by exposure to TNF-α and IFN-γ, while MIT-001 recovered this value by improving SRC to an almost equal value of the control hPD-MSCs (Figure 3E). Meanwhile, despite the proton leak and the causes of ROS production having no statistical significance, the data showing the actual cellular ROS revealed a nearly 40% higher level of ROS in the TNF-α/IFN-γ-exposed hPD-MSCs compared to the control and MIT-001-treated groups (Figure 3F). Collectively, these data demonstrate that MIT-001 reduces excessive mitochondrial oxygen consumption, which inhibited an increase in ROS production and MSC senescence in a homeostatic way.

### 2.4. MIT-001 Ameliorated Inflammatory Phenotype of TNF-α/IFN-γ-Exposed hPD-MSCs

**Figure 4:**
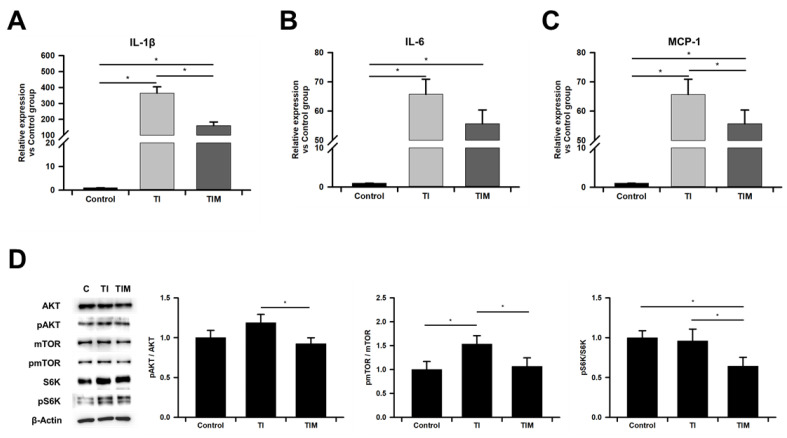
MIT-001 improved senescent phenotypes of hPD-MSCs. (**A**–**C**) Real-time qPCR results demonstrating the representative SASP expression levels of IL-1β, IL-6, and MCP-1. (**D**) Western blot analysis and phosphorylation ratios determined from the band intensities of anti-pAKT/anti-AKT, anti-pmTOR/anti-mTOR, anti-pS6K/anti-S6K, and the housekeeping protein β-actin in the control, TNF-α/IFN-γ -exposed, and TNF-α/IFN-γ with MIT-001-treated hPD-MSCs. C: control, TI: TNF-α + IFN-γ, TIM: TNF-α + IFN-γ + MIT-001. Data are presented as the mean ± SEM (*n* = 3). Statistical significance was determined by a one-way ANOVA. * *p* < 0.05.

MSCs exposed to TNF-α and IFN-γ have been known to obtain pro-inflammatory phenotypes by secreting the senescence-associated secretory phenotype (SASP) and damage-associated molecular pattern (DAMP), which induce further inflammation in a feedback loop [17,32,33]. Elevated expression of SASP has been widely reported in MSCs primed with either TNF-α or IFN-γ or both. Consistently, we also confirmed that TNF-α and IFN-γ significantly increased the expression of SASPs, including IL-1β, IL-6, and monocyte chemotactic protein-1 (MCP-1) (Figure 4A–C). MIT-001 treatment had no large effect on IL-6. However, IL-1β and MCP-1 were significantly down-regulated by MIT-001 by about 55 and 15 percent, respectively.

Next, we analyzed the phosphorylation-induced activation ratio of AKT/mTOR/S6K, which refers to the mTORC1 pathway. mTORC1 activation has been known to not only trigger the senescence-associated phenotype of MSCs but also compromise their quiescence and stemness. Among the three molecules involved in the mTORC1 pathway, the phosphorylation ratio of mTOR was significantly up-regulated via exposure to TNF-α and IFN-γ, while its representative upstream (AKT) was hardly affected. In addition, despite a similar value for S6K phosphorylation ratio, substantially increased quantities of both S6K and pS6K are observed in Figure 4D. Importantly, the phosphorylation ratios of AKT, S6K, and mTOR were significantly down-regulated by about 25, 40, and 30 percent, respectively, after MIT-001 treatment compared to the TNF-α/IFN-γ-exposed hPD-MSCs. Collectively, these data reveal that MIT-001 prevents the senescence phenotype of TNF-α/IFN-γ-exposed MSCs by inhibiting the expression of SASP factors and activation of the mTORC1 pathway, a major cause of cellular senescence.

## 3. Discussion

In this study, we identified the altered mitochondrial phenotype of hPD-MSCs and its enhancement using a mitochondrion-targeted small molecule ROS scavenger, MIT-001. In a healthy state, MSCs have a fibroblast-like shape with a low level of metabolism [13,15,28]. The fibroblast-like spindle shape of MSC has been regarded as the best visual marker in that MSCs begin to lose their original morphology as due to either aging or a metabolic switch to the activation state, resulting in increased morphologic heterogeneity and abnormal differentiation [13,15]. Here, we also demonstrated that hPD-MSCs exposed to TNF-α and IFN-γ lose their spindle-like shape, exhibiting abnormal morphology, whereas the control and TNF-α/IFN-γ-exposed hPD-MSCs with MIT-001 treatment showed thinner and more spindle-like shapes in their morphology. Given that MSC morphology implicates not only the metabolic synthesis/degradation rate balance but also the properties and fate of stem cells [28,34,35], the altered morphology and well-maintained state after MIT-001 treatment suggest that MIT-001 enhanced the MSC phenotype by enhancing a specific feature related to homeostasis. This recovered morphology of hPD-MSCs is further supported by the organized structure of the microfilaments. The cytoskeletal network is composed of three factors: microtubules, interfilaments, and microfilaments. However, among the three components of the cytoskeleton, the organization of microfilaments has been determined to be the most strongly related to stem cell fate and maintenance, while microtubules and interfilaments provide only minor contributions [21,22,23]. Several studies revealed that undifferentiated MSCs have thin, parallel microfilament bundles extending across the long cell axis, whereas differentiated cells exhibit disturbed microfilaments with diffused, thicker, and crisscrossed fibers [21,23]. Consistently, we also demonstrated altered microfilament structures in the TNF-α/IFN-γ-exposed hPD-MSCs, with disrupted organization vertical to the long cell axis, followed by amelioration upon MIT-001 treatment.

Having identified the enhanced visual markers of hPD-MSCs, we further analyzed the representative genes for cell proliferation and stemness. As expected, TNF-α and IFN-γ drastically hampered cell proliferation and stemness by inhibiting the cell cycle progressor CCNA2 and stemness factor NANOG, and up-regulated cell cycle inhibitor P16, while MIT-001 significantly recovered the expression of P16 and NANOG. However, future studies on more specific mechanisms related to stemness are required since the expression of OCT4, another stemness marker, was markedly increased after TNF-α/ IFN-γ exposure. Similar to our results, Wang et al. showed the knock-out or inhibition of either TNF-α or IFN-γ restored MSC proliferation and osteogenic differentiation [5].

To further investigate the MSC phenotype, we next investigated mitochondrial homeostasis with or without MIT-001 treatment. Priming TNF-α/IFN-γ induced abnormal mitochondrial distribution extending perpendicular to the cell axis and significantly augmented the expression of both the mitochondrial fission gene (DRP1) and fusion genes (MFN1 and MFN2) compared to the control group. However, MIT-001 treatment protected the mitochondrial distribution which is parallel to the long cell axis and significantly decreased both mitochondrial fission and fusion genes, as did the control group of hPD-MSCs. In general, the expression of mitochondrial fusion and fission genes is inversely proportional. For example, Fu et al. comprehensively documented that decreased mitochondrial fission and increased fusion can be observed when stem cells are differentiated or aged [36]. Forni et al. also reported up-regulated mitochondrial fusion during the adipogenic or osteogenic differentiation of MSCs, while chondrogenic differentiation requires increased expression of mitochondrial fission-related genes [37]. Given that most studies demonstrated only the inverse proportion of mitochondrial fission and fusion, the simultaneous increase and decrease in DRP1 and MFN1/2 under TNF-α/IFN-γ exposure provides a new perspective on the more systemic landscape of mitochondrial dynamics in impaired stem cells and requires more detailed research [32,37,38,39].

Mitochondrial distribution can also be considered a crucial marker for MSC differentiation [38]. Quinn et al. reported that undifferentiated MSCs exhibit a perinuclear arrangement of the mitochondria around the nucleus and are uniformly distributed in the cytoplasm [40]. Moreover, mitochondrial distribution was observed to be different depending on whether the mitochondria differentiated. Similarly, we also demonstrated that MIT-001 promoted mitochondrial distribution such that the mitochondria were distributed around the nucleus, rather than outside the cytoplasm. Although the more precise correlation between mitochondrial distribution and the MSC phenotype needs to be studied further, the improved morphology of the mitochondria and the cellular shape suggest the promising homeostatic effect of MIT-001 in response to inflammatory conditions. Indeed, many studies have explored the link between mitochondria and the cytoskeleton. For instance, microfilaments are known to regulate the short-range movement of mitochondria, distributing them to areas where greater energy or metabolic functions are required [41,42]. Moore et al. emphasized that dynamic interactions between the mitochondria and the cytoskeleton are critically important to maintain the mitochondrial network’s structure and function [42].

After characterizing the altered mitochondrial phenotype, we confirmed a decrease in oxygen consumption resulting in a sequential reduction in ATP production and proton leakage. MSCs have a low oxygen consumption rate to prevent excessive ROS production and maintain their homeostasis, which ensures a quiescent state. However, the level of mitochondrial respiration increases with aging to meet the higher energy demands of senescent or differentiated MSCs [13]. The up-regulated level of mitochondrial respiration inevitably accompanies ROS production, which damages the genome, other organelles, proteins, and the mitochondria themselves. Hence, our data showing a decrease in oxygen consumption and mitochondrial respiration down to the control-group level indicate that MSC maintenance and quiescence were markedly enhanced by MIT-001. Importantly, these enhanced mitochondrial features are further supported by recovery in SRC. SRC refers to the amount of extra ATP that can be produced by mitochondrial respiration in case of a sudden increase in energy demand to protect mitochondrial homeostasis against environmental stresses [43]. Generally, stem cells have low levels of SRC due to their quiescent mitochondrial profile compared to differentiated or senescent cells [43,44]. However, several studies have reported that pathological conditions such as inflammation and mitochondrial dysfunction further reduce the SRC of MSCs. For instance, MSCs derived from adults with coronary artery disease exhibited lower levels of SRC in parallel with oxygen consumption and ATP production [45]. Ye et al. also demonstrated that BM-MSCs in ankylosing spondylitis displayed significantly decreased SRC compared to healthy MSCs [46]. That is, our data demonstrating decreases in basal respiration, ATP production, and ROS levels followed by increases in SRC indicate that MIT-001 promotes mitochondrial homeostasis, rather than provokes mitochondrial damage or dysfunction. Collectively, MIT-001 improved the compromised mitochondrial phenotypes, including the mitochondrial morphology and metabolism caused by inflammatory cytokines TNF-α and IFN-γ. Similarly, numerous studies have demonstrated the enhancement of MSC functions by blocking oxygen consumption and ROS production [12,47,48].

To further specify the connectivity between enhanced mitochondrial homeostasis and actual MSC phenotypes, we also investigated the SASP secretion and mTORC1 pathway regulating senescence of MSCs. The priming inflammatory cytokines TNF-α and IFN-γ are known to temporarily improve the anti-inflammatory roles of MSCs. On the other hand, such pretreatment reportedly activates MSC metabolism, resulting in not only a loss of intrinsic homeostasis but also increased production of senescence-associated secretory phenotypes (SASP) such as pro-inflammatory cytokines, growth factors, and proteases [17,32,33]. For instance, Zhao et al. recently showed that TNF-α significantly augments the expression and secretion of inflammatory cytokines IL-1β, IL-6, MMP9, and MCP-1 and their attenuation by inhibiting the ER-stress-activated JNK pathway [49]. Neri et al. also documented that the mitochondrial dysfunction caused by MSC senescence contributes to SASP and ROS production [33]. In the same context, our results reveal that MIT-001 can significantly reduce a very high value of SASP expression, especially in IL-1β and MCP-1. This result suggests that the mitochondrial dysfunction-dependent pathway is included in the impaired phenotypes of hPD-MSCs exposed to TNF-α/IFN-γ. In addition, we also examined the mTORC1 pathway, the main signal pathway for SASP production. MIT-001 inhibited the mTORC1 pathway by reducing the phosphorylation ratio of mTOR, its upstream molecule AKT, and the downstream molecule S6K. Including SASP production, mTORC1 has been known to play a variety of roles in autophagy, molecular synthesis, and senescent growth. Khorraminejad-Shirazi et al. determined that the inhibition of mTORC1 by antioxidants or the activation of 5′ adenosine monophosphate-activated protein kinase (AMPK) enhanced the function of MSC. In our previous study, we also reported that the mitochondrial open reading frame of the 12S rRNA-c (MOTS-c) enhanced the homeostasis of hPD-MSCs by activating AMPK and inhibiting the mTORC1 pathway, thereby promoting mitochondrial function and quiescence [50]. Moreover, the inhibition of mTORC1 has been considered a promising clinical strategy due to its ability to improve the autophagy and functionality of tissues and organs [11]. Gharibi et al. attenuated age-related changes in MSCs by inhibiting AKT/mTOR and S6K [48]. Meanwhile, although it is not understood whether AKT and mTOR are direct target molecules for MIT-1, Park et al. reported that NecroX-5, another family molecule of MIT-001 (previously known as NecroX-7), inhibited AKT via a reduction in the intracellular calcium levels in mouse breast cancer 4T1 cells and human breast cancer HCC70, MDA-MB-231, and MDA-MB-453 cells [51].

In conclusion, our study reveals that MIT-001 prevented the senescent phenotypes of impaired hPD-MSCs caused by exposure to TNF-α and IFN-γ in vitro. Both major inflammatory cytokines excessively activate mitochondrial dynamics and metabolisms, resulting in increased ROS levels, SASP production, morphological heterogeneity, and an irregular cytoskeletal structure. Importantly, these phenotypes were significantly improved by MIT-001, which has been studied to prevent degenerative and inflammatory diseases such as bone destruction and graft versus host disease. However, more sophisticated study should be conducted to evaluate the clinical potential of MIT-001. For example, in the systemic body, MSCs are exposed to a variety of pro-inflammatory cytokines beyond TNF-α and IFN-γ. Moreover, most chronic inflammation-induced phenotypic changes are progressed before actually detecting the inflammation-related lesions or diseases. Therefore, studies investigating whether the MIT-001 can protect the already altered phenotypes of MSCs and tissues are essentially required in the future. Taken together, these results further underscore the clinical potential of MIT-001 as a drug for the enhancement of MSC phenotypes, indicating that MIT-001 not only represents a strategy for stem cell therapy but also has promising clinical use in vivo. Moreover, this study also suggests a new perspective to treat inflammatory diseases or inflammaging on a more fundamental level by enhancing MSC homeostasis, which plays a role in tissue homeostasis and may promote healthy aging and longevity.

## 4. Materials and Methods

### 4.1. Cell Culture and MIT-001 Treatment

hPD-MSCs harvested from the inner side of the chorioamniotic membrane of the placenta were donated by Dr. Gi Jin Kim (Cha University, Seongnam, Korea). Cells were cultured in an alpha-Minimum Essential Medium (Cytiva, Marlborough, MA, USA) supplemented with 10% fetal bovine serum (Thermofisher, Waltham, MA, USA), 1% penicillin/streptomycin (Thermofisher, Waltham, MA, USA), 25 ng/mL human fibroblast growth factor-4 (Peprotech, Rocky Hill, NJ, USA), and 1 µg/mL heparin (Merck, San Francisco, CA, USA). A total of 1 × 10^5^ hPD-MSCs were seeded on a cell culture dish (90 × 20 mm) (SPL, Pocheon, Korea) and kept in an incubator containing 5% CO_2_ at 37 °C. Cells were passaged every 4 days when they reached 70–80% confluency. Expanded cells were harvested at passages 12 or 13. In passages for sampling, the hPD-MSCs were cultured in a medium containing 2.5 ng/mL TNF-alpha (Peprotech), 25 ng/mL IFN-gamma (Peprotech), and with or without MIT-001 (C24H29N3O3S; patent no. KR2008-0080519; previously known as NecroX-7; kindly donated by MitoImmune Therapeutics Inc., Korea) for 24 h. Viable cells were manually counted using Trypan blue (Merck), which distinguishes between live and dead cells.

### 4.2. Fluorescence Imaging of Phalloidin-Stained Cells

A total of 5 × 10^4^ hPD-MSCs were seeded in a 12-well plate (SPL). After 24 h, the hPD-MSCs were fixed using 4% PFA and then permeabilized with 1% Triton X solution. Cells were treated for 20 and 5 min, respectively, with Phalloidin, which labels the cellular F-actin structure, and Hoechst 33342, which labels nuclei. Stained cells were imaged using an inverted fluorescence microscope equipped with a DS-5i camera (Eclipse Ti-Ul; Nikon, Tokyo, Japan).

### 4.3. Reverse Transcription and Real-Time qPCR

The total RNA of the harvested hPD-MSCs was extracted using an easy-spin Total RNA Kit (iNtRON, Seong-nam, Korea). The RNA concentration was quantified using a microplate spectrometer (Epoch™ Microplate Spectrophotometer, BioTek, Winooski, VT, USA) and adjusted to 100 ng/µL. RNA was reverse-transcribed into cDNA with RT PreMix(dT20) (Bioneer, Seoul, Korea) using a SimpliAmp Thermal Cycler (Life Technologies, Carlsbad, CA, USA). Real-time qPCR was performed with a real-time PCR machine (CFX Connect, Bio-Rad, Hercules, CA, USA), SYBR Green Supermix (Bio-Rad), and primers (Table S1). All experiments were repeated three times for the statistical analysis.

### 4.4. Western Blotting

Proteins were extracted with a Pro-Prep protein lysis buffer (iNtRON) and quantified with a protein quantification assay kit (BIOMAX, Seoul, Korea). Samples were then boiled in 4× Laemmli sample buffer (Bio-Rad) containing 2-mercaptoethanol (Bio-Rad). The protein was loaded into an 8% sodium dodecyl sulphate polyacrylamide gel and electrophoresed at 60 V for 30 min followed by 120 V for 1 h. The proteins were transblotted onto a nitrocellulose membrane (Bio-Rad) at 400 mA for 90 min. The membrane was incubated with a blocking buffer (TBS-T containing 4% bovine serum albumin) for 1 h followed by a primary antibody solution overnight at 4 °C. Anti-AKT1 (Thermofisher), anti-pAKT1 (Invitrogen, IL, USA), anti-mammalian target of Rapamycin (Cell Signaling, Danvers, Massachusetts, USA), anti-pmTOR (Abcam, Cambridge, MA, USA), anti-ribosomal protein S6 kinase beta-1 (Cell Signaling), anti-pS6K (Cell Signaling), and anti-β-actin (Thermofisher) primary antibodies were used. The blotted membrane was incubated with an anti-mouse (Thermofisher) or anti-rabbit (Thermofisher) secondary antibody for 1 h at room temperature. Immunoreactive bands were detected using an enhanced chemiluminescence detection reagent (ClarityTM Western blot substrate, Bio-Rad). Images of the bands were acquired using ImageSaver Version 6 (ATTO, Tokyo, Japan). The intensity of each band was analyzed with the CA4 analyzer software (ATTO). The experiment was repeated three times under the same conditions with different samples.

### 4.5. Oxygen Consumption Rate (OCR) Measurements of Cells

The OCR was measured using a Seahorse XFp analyzer (Agilent, Santa Clara, CA, USA). Prior to the assay, a total of 10,000 cells/well were seeded into a Seahorse XFp Cell Culture Miniplate and incubated with TNF-alpha (Peprotech), IFN-gamma (Peprotech) and with or without MIT-001 for 24 h. The growth medium was then changed to a Seahorse XF Base Medium containing 2 mM L-glutamine, 5.5 mM D-glucose, and 1 mM sodium pyruvate, according to the composition of α-MEM. During the assay, cells were treated with 1 µM oligomycin, 0.5 µM FCCP, and 0.5 µM rotenone/antimycin A. The OCR was measured for 2 min after mixing for 3 min, which was repeated 12 times. The basal OCR was normalized according to the protein concentration determined by the BCA assay. The results were then obtained using the manufacturer’s software.

### 4.6. Mitochondrial Function Analysis of Cells

To investigate the mitochondrial membrane potential and mitochondrial mass, hPD-MSCs were co-stained with MitoSpy Orange CMTMRos (BioLegend, San Diego, CA, USA), which labels mitochondria based on their membrane potential, and MitoSpy Green FM (BioLegend), which labels mitochondria independent of their membrane potential. After treatment with or without MOTS-c for 72 h, hPD-MSCs were passaged on gelatin-coated cover glasses (Sigma, St.Louis, Missouri, USA) in a 12-well plate (SPL) and stained after 24 h. hPD-MSCs were incubated in α-MEM containing 250 nM MitoSpy Green FM and 250 nM MitoSpy Orange CMTMRos for 30 min. Stained hPD-MSCs were fixed with 4% paraformaldehyde (Alfa Aesar, Ward Hill, Massachusetts, USA) for 5 min and then stained with 1 µg/mL Hoechst 33342^®^ (Thermofisher) for 5 min at room temperature. Finally, the cover glasses were mounted on glass slides with a mounting solution (VectaShield, Thermofishe). Samples were imaged with a confocal microscope (LSM880; Zeiss, Oberkochen, Germany). Fluorescence intensities in confocal images were quantified using the ZEN 2018 Black and Blue edition software (Zeiss).

### 4.7. Reactive Oxygen Species (ROS) Assay

A total of 5 × 10^3^ hPD-MSCs were seeded in each well of a 96-well black-microplate (SPL, 33196) and cultured for 24 h. To investigate the level of ROS, cells were washed and incubated in pre-warmed Hanks’ balanced salt solution (14025092, Thermofisher) containing 50 μM H2DCFDA (D399; Thermofisher) for 30 min. Fluorescence intensity at 555 nm was measured with excitation at 488 nm using a Multi-Mode Micro-Plate Reader (Flextation 3, Molecular Devices, Silicon Valley, CA, USA).

### 4.8. Statistical Analysis

All experiments were repeated more than three times. Statistical analysis was performed using the Sigma-plot 12.5 software. A one-way ANOVA was used to determine statistical significance, followed by a post-hoc Holm–Sidak test. The significance level was set at * *p* < 0.05.

## 5. Patents

The molecule “MIT-001” is patented: KR2008-0080519.

## Supplementary Materials

The following are available online at https://www.mdpi.com/article/10.3390/ijms22105062/s1.
