# Supplementary material for: MIT-001 Restores Human Placenta-Derived Mesenchymal Stem Cells by Enhancing Mitochondrial Quiescence and Cytoskeletal Organization
Source: Int J Mol Sci. 2021 May 11;22(10):5062. doi: 10.3390/ijms22105062 (PMC8151078; doi:10.3390/ijms22105062)
Supplement: Supplementary file 1 [file ijms-22-05062-s001.zip › Primer table.pdf]

Table S1. Primer set for real-time qPCR

Primer name	Gene code	Forward primer<br>Backward primer	PCR condition	Product size
**hβ-actin**	X00351.1	5’-ACAATGTGGCCGAGGACTTT -3’<br>5’-TGTGTGGACTTGGGAGAGGA -3’	58℃	104
**hCCNA2**	NM_001237.5	5’-TGCTGACCCATACCTCAAGT-3’<br>5’-TGACTGTTGTGCATGCTGTG-3’	58℃	211
**hP16**	NM_000077.4	5’- CGGAGGAAGAAAGAGGAGGG-3’<br>5’- GGCCTCCGACCGTAACTATT-3’	58℃	252
**hOCT4**	NM_002701.6	5’-GGAGTTTGTGCCAGGGTTTT-3’<br>5’-ACTTCACCTTCCCTCCAACC-3’	58℃	238
**hNANOG**	NM_024865.4	5’-AAGGTCCCGGTCAAGAAACA-3’<br>5’-TCTGCGTCACACCATTGCTA-3’	58℃	175
**hDRP1**	NM_001278464.1	5’- TCCATGAGACTTTTGGGCGA-3’<br>5’- TTGCCGCTTCACCAGTAACT-3’	58℃	152
**hMFN1**	NM_033540.3	5’- ACAAGGTGAATGAGCGGCTT-3’<br>5’- TCCACCAAGAAATGCAGGCA-3’	58℃	136
**hMFN2**	NM_014874.4	5’- ACCGTGATCAATGCCATGCT-3’<br>5’- TGGTTCACAGTCTTGGCACT-3’	58℃	155
**hIL-1β**	NM_000576.3	5’- TCAGCACCTCTCAAGCAGAA-3’<br>5’- TCCACATTCAGCACAGGACT-3’	58℃	136
**hIL-6**	NM_000600.5	5’- AATAACCACCCCTGACCCAA-3’<br>5’- TGCTACATTTGCCGAAGAGC-3’	58℃	153
**hMCP-1**	NM_002982.4	5’- GCAAGTGTCCCAAAGAAGCT-3’<br>5’- TCCTGAACCCACTTCTGCTT-3’	58℃	85
