# Supplementary material for: MIT-001 Restores Human Placenta-Derived Mesenchymal Stem Cells by Enhancing Mitochondrial Quiescence and Cytoskeletal Organization
Source: Int J Mol Sci. 2021 May 11;22(10):5062. doi: 10.3390/ijms22105062 (PMC8151078; doi:10.3390/ijms22105062)
Supplement: Supplementary file 1 [file ijms-22-05062-s001.zip › Supplement Figure text.pdf]

Figure S1. Representative bright-field images of hPD-MSCs with or without TNF-α/IFN-γ and MIT-001. The concentration of MIT-001 were tested from 0.1 μM to 5 μM and DMSO group. Black scale bar, 200 μm. . C: Control, TI: TNF-α + IFN-γ TIM: TNF-α + IFN-γ + MIT-001.
