# Supplementary figures and images for: MIT-001 Restores Human Placenta-Derived Mesenchymal Stem Cells by Enhancing Mitochondrial Quiescence and Cytoskeletal Organization
Source: Int J Mol Sci. 2021 May 11;22(10):5062. doi: 10.3390/ijms22105062 (PMC8151078; doi:10.3390/ijms22105062)

**Figure S1. MSC phenotype by concentration**

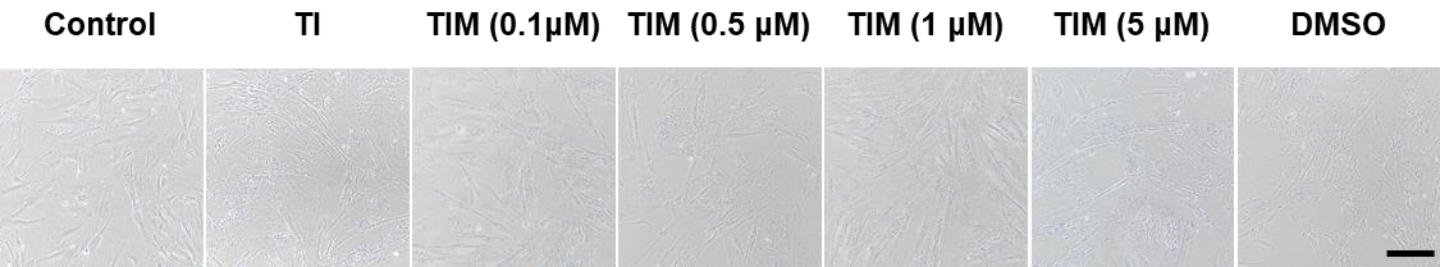

Supplement: Supplementary file 1 [file ijms-22-05062-s001.zip › Supplement Figure S1.pdf]
